# Radioiodination of rat hepatoma-specific antigens and retention of serological reactivity.

**DOI:** 10.1038/bjc.1980.133

**Published:** 1980-05

**Authors:** D. Hannant, J. G. Bowen, M. R. Price, R. W. Baldwin

## Abstract

Papain-solubilized tumour-specific antigens from the aminoazo dye-induced rat hepatoma D23 were purified by a combination of lectin affinity and immunoadsorbent column chromatography. Isolated antigens were radio-iodinated using three procedures and analysed for their reaction with specific antibodies in syngeneic immune sera by double-antibody co-precipitation tests and by the rebinding of labelled antigens to specific and non-relevant antibodies immobilized on Sepharose-4B. Soluble hepatoma D23-specific antigens were labile to radiolabelling, and for optimal retention of serological reactivity it was necessary to protect the antigenic determinant by performing the chloramine T method of iodination with antigen bound to the immunoadsorbent followed by elution from the solid phase with 3M NaSCN. Immunoadsorption chromatography indicated that one consequence of radiolabelling hepatoma D23-specific antigen with 125I was a reduction in the affinity of the labelled antigen for its syngeneic specific antibody.


Br. J. Cancer (1980) 41, 716

### RADIOIODINATION OF RAT HEPATOMA-SPECIFIC ANTIGENS AND RETENTION OF SEROLOGICAL REACTIVITY

D. HANNANT, J. G. BOWEN*, M. R. PRICE AND R. W. BALDWIN

*From the Cancer Research Campaign Laboratories, University of Nottingham, University Park, Nottingham NG7 2RD*

Received 25 October 1979 Accepted 16 January 1980

**Summary.—Papain-solubilized tumour-specific antigens from the aminoazo dye-induced rat hepatoma D23 were purified by a combination of lectin affinity and immunoadsorbent column chromatography. Isolated antigens were radio-iodinated using three procedures and analysed for their reaction with specific antibodies in syngeneic immune sera by double-antibody co-precipitation tests and by the rebinding of labelled antigens to specific and non-relevant antibodies immobilized on Sepharose-4B. Soluble hepatoma D23-specific antigens were labile to radiolabelling, and for optimal retention of serological reactivity it was necessary to protect the antigenic determinant by performing the chloramine T method of iodination with antigen bound to the immunoadsorbent followed by elution from the solid phase with 3M NaSCN. Immunoadsorption chromatography indicated that one consequence of radiolabelling hepatoma D23-specific antigen with $^{125}$I was a reduction in the affinity of the labelled antigen for its syngeneic specific antibody.**

AMINOAZO DYE-INDUCED rat hepatomas are characterized by the expression of individually distinct tumour-specific antigens demonstrable serologically or by tumour transplant rejection (Baldwin, 1973; Moore, 1978). The serologically defined tumour-specific antigen associated with the rat hepatoma D23 is expressed on an integral plasma-membrane glycoprotein, and the antigenically active, water-soluble glycopeptide released by limited proteolysis with papain displays a molecular weight of $\sim$55,000 (Price & Baldwin, 1977). There is currently a need to develop objective assays for the detection of these antigens, so that the chemical analysis of these products may be conducted on a more quantitative basis. Radio-isotopic antiglobulin tests have revealed tumour-specific antigens on rat hepatomas by the detection of specific antibodies in syngeneic immune sera binding to surface-membrane antigens on dispersed tumour cells (Al-Sheikly *et al.*, 1979). Although antibody binding was demonstrable at serum dilutions as low as 1/250, these reactions are not sufficiently strong to allow modification of the test as a routine assay for acellular antigens by determining their capacity to inhibit antibody-binding reactions.

The present study was therefore undertaken to establish the appropriate conditions for radiolabelling lectin affinity and immunoadsorbent-purified, papain-solubilized hepatoma-associated antigens with the retention of serological activity, so that cell-free radioimunoassays might be developed for the routine quantitation of these components.

### MATERIALS AND METHODS

### *Rats, tumours and sera*

Inbred WAB/Not (Nottingham Cancer Research Campaign subline of WAB) rats were maintained by single-line brother-sister mating. Hepatomas D23 and D192, induced by

* Present address: The Boots Co. Ltd., Research Department R3, Pennyfoot Street, Nottingham.

oral administration of 4-dimethylaminoazobenzene, sarcoma Mc7, induced by s.c. injection of 3-methyl-cholanthrene, and the spontaneously arising mammary carcinoma Sp4, were maintained by serial s.c. passage in WAB/Not rats. Hepatoma D23- and D192-immune serum, sarcoma Mc7-immune serum and mammary carcinoma Sp4-immune serum were prepared in syngeneic rats by weekly i.p. injections of $2 \times 10^7$ $\gamma$-irradiated (15,000 R) tumour cells. Serum donors were bled by cardiac puncture under ether anaesthesia, and the serum pools were collected and stored at $-20°C$. For the isolation of hepatoma D23 antigen, cells from the ascitic variant of D23 were injected i.p. in $\gamma$-irradiated (450 R) WAB/Not rats to prevent the coating of tumour cells by IgG antibody (Robins, 1975).

*Solubilization and purification of hepatoma D23 specific antigen*

Hepatoma D23 ascites cells were harvested from the peritoneal cavity of tumour donors and the cells washed $\times 3$ by centrifugation with Hanks's balanced salt solution (HBSS). Papain (18 mg) (Sigma Chemical Co., Kingston upon Thames) was added to $1 \cdot 8 \times 10^{10}$ viable tumour cells suspended at $10^8$/ml in HBSS containing 5mM L-cysteine, and hydrolysis was allowed to proceed for 45 min at 37°C. The cell suspension was rapidly cooled on ice, the cells were sedimented by centrifugation at 400 *g* for 15 min and the supernatant was centrifuged at 105,000 *g* for 60 min to ensure solubility. Papain was removed by ion-exchange chromatography on DEAE–cellulose, as previously described by Baldwin *et al.* (1973*a*). The soluble extract was then subjected to a series of chromatographic procedures as summarized in Table I. Briefly, the antigenic glycopeptide fraction bound to Concanavalin A-linked Sepharose-4B (Pharmacia Ltd., Uppsala, Sweden) was eluted with 0·2M $\alpha$-methylmannoside according to the manufacturer's recommendations. This fraction was passed over a column of immobilized Ig from a sheep anti-rat IgG antiserum linked to CNBr-activated Sepharose 4B (Pharmacia Ltd.) and the unbound fraction was applied to a column of Sepharose-4B-conjugated IgG from syngeneic WAB/Not anti-hepatoma D23 immune serum. (All immunoadsorbents were prepared by conjugating protein to CNBr-activated Sepharose-4B at 10 mg protein/g dry weight of activated Sepharose, and $>90\%$ of protein was regularly conjugated to the solid phase). After washing the column with phosphate-buffered saline, pH 7·3, (PBS) to remove non-bound material, the column was washed with 0·1M glycine-NaOH, 0·5M NaCl (pH 9·0) to eliminate non-specifically bound material (Zöller & Matzku, 1976). Hepatoma D23 antigen was dissociated from the immunoadsorbent by application of 3M NaSCN, and the eluate was desalted by passage over Sephadex-G25 (Pharmacia Ltd.) equilibrated with PBS. The full details of these preparative procedures have been given elsewhere (Baldwin *et al.*, 1973*a*, *b*; Preston & Price, 1977; Bowen & Baldwin, 1979; Price *et al.*, 1979) and such antigen preparations were characterized both by the specific inhibition of indirect membrane immunofluorescence reactions and by their capacity to induce tumour-specific antibody in treated rats.

*Radioiodination of hepatoma D23 antigens*

The following procedures were used in an attempt to prepare radiolabelled hepatoma D23-specific antigens retaining serological reactivity with antibodies in syngeneic WAB/Not antihepatoma D23 immune serum. All iodinations were performed on the same batch of purified antigens.

*Direct labelling of soluble antigens.*—A modified chloramine T method (McConahey & Dixon, 1966) was adopted to label 10 $\mu$g aliquots of protein with $^{125}$I (Na $^{125}$I, Radiochemical Centre, Amersham, Bucks.) and reaction conditions were varied to give specific activities in the range 0·1–15 $\mu$Ci/$\mu$g protein, protein concentrations being determined by the Lowry method. At the highest level of labelling used, the calculated number of iodine atoms per molecule of antigen did not exceed unity.

*Conjugation of $^{125}$I-labelled tyrosine to soluble antigens.*—Tyrosine (Tyr) (100 ng) was labelled with 500 $\mu$Ci $^{125}$I by a modification of the lactoperoxidase-catalysed reaction described by Phillips & Morrison (1971). The reaction was performed in 0·05M sodium cacodylate buffer (pH 5·6) using 5 ng lactoperoxidase (Sigma Chemical Co.) and a total of 600 ng $H_2O_2$ was added to the reaction mixture (55 $\mu$l) over a period of 28 min. The $^{125}$I-labelled Tyr was coupled to 5 $\mu$g of immunoadsorbent-purified hepatoma D23 antigen by the formation of amide bonds with 10 $\mu$g EDC (1-ethyl-3(3-dimethylamino-propyl)-carbodiimide-hydrochloride, Sigma Chemical

Co.). Coupling was allowed to proceed at pH 5·6 in a total reaction volume of 85 μl at 4°C for 18 h. The $^{125}$I-Tyr coupled hepatoma D23 antigen was separated from free $^{125}$I and unreacted $^{125}$I-Tyr by chromatography on Sephadex-G150 equilibrated with PBS containing 0·05% bovine serum albumin (BSA, Sigma Chemical Co.). The specific activity of the labelled antigen was ~20 μCi/μg protein, about 90% of radioactivity being precipitable by 10% trichloracetic acid (TCA).

*Indirect labelling of soluble antigen immobilized on an affinity substrate.*—Briefly, immunoadsorbent-purified hepatoma D23 antigen (~10 μg protein) was incubated with Sepharose-4B-linked IgG from WAB/Not anti-D23 immune serum (0·1 ml of 50% suspension). The pellet was washed and reacted with Na $^{125}$I (500 μCi) using the reactants described by McConahey & Dixon (1966). Elution of radiolabelled antigen was performed with 3M Na SCN after washing the pellet with 0·1M glycine-NaOH, 0·5M NaCl (pH 9·0). Radio-labelled hepatoma D23 antigen was chromatographed on Sephadex-G25 equilibrated in PBS containing 0·05% BSA to effect buffer exchange and 85±3% (6 determinations) of radioactivity was precipitable with 10% TCA.

*Co-precipitation radioimmunoassay*

Polystyrene round-bottomed tubes (9·5 × 63·5 mm) were pre-coated with 10% foetal calf serum (FCS) in PBS. Radiolabelled antigen preparations were dispensed in 10μl aliquots (0·7–1·8 ng protein/tube) and incubated with 20 μl of WAB/Not anti-hepatoma D23 immune serum, other immune serum or normal WAB/Not serum for 18 h at 4°C. The precipitating antiglobulin reagent (sheep anti-rat IgG or goat anti-rat IgG) was added at a dilution previously established to precipitate 100% rat IgG by standard quantitative precipitation tests using $^{125}$I-labelled WAB/Not IgG. The reactants were incubated at room temperature for 90 min and 1·0 ml PBS containing 0·05% BSA was added to each tube before centrifugation at 1000 *g* for 10 min. Supernatants were discarded and pellets were washed with PBS–BSA ×3 centrifugation at 1000 *g* for 10 min before determining their content of radioactivity with an LKB-Wallac Gamma Counter. Results were recorded in terms of % co-precipitation of $^{125}$I-labelled hepatoma D23 antigen, where % co-precipitation

$$= \frac{\text{(ct/min in precipitate} - \text{ct/min in medium control}}{\text{(ct/min precipitable with 10\% TCA} - \text{ct/min in medium control}} \times 100$$

*Rebinding of $^{125}$I-labelled soluble antigen to immunoadsorbents.*—Radio-iodinated antigens (100 μl at 5–10 μg/ml) were applied to columns (~2 ml volume) of Sepharose-4B-linked IgG from hepatoma D23 or D192-immune serum. These columns were washed and eluted with the same buffers and dissociating agent as described for the preparation of antigen by specific immunoadsorption, and the distribution of radioactivity in unbound fractions and 3M NaSCN-eluted fractions was determined.

### RESULTS

The chloramine T method of McConahey & Dixon (1966) for the trace iodination of proteins was efficient at radiolabelling papain-solubilized hepatoma D23 antigen purified by lectin affinity and immunoadsorbent column chromatography accord-

TABLE I.—*Chromatographic purification of hepatoma D23-specific antigen*

*Papain solubilization of surface-membrane glycoproteins*
1·8 × 10$^{10}$ hepatoma D23 ascites cells treated with 18·0 mg papain, 37°C, 45 min and soluble extract collected

↓

*Removal of papain*
Ion-exchange chromatography on DEAE cellulose; membrane glycoproteins eluted with 0·5M NaCl in Tris-phosphate (pH 4·5)

↓

*Lectin affinity chromatography*
Material bound to Sepharose-4B-linked Con A and eluted with 0·2M α methylmannoside

↓

*Affinity-subtraction chromatography*
Non-bound material collected from Sepharose-4B-linked sheep anti-rat IgG

↓

*Specific affinity chromatography*
Material applied to Sepharose-4B-linked syngeneic anti-hepatoma D23 IgG. Non-specifically bound material removed with pH 9·0 buffer. Antigen eluted with 3M NaSCN

↓

*Hepatoma D23-specific antigen*

(~20 μg protein)

ing to the procedure in Table I. About 40% of added $^{125}I$ was regularly recovered in labelled antigen preparations when the reaction conditions were varied to achieve specific activities between 0·1 and 15 $\mu$Ci/$\mu$g protein. The results of assaying one of these preparations (sp. act. 5 $\mu$Ci/$\mu$g) in co-precipitation tests with syngeneic rat sera (hepatoma D23-immune serum, sarcoma Mc7-immune serum and normal rat serum) and the heterologous anti-globulin reagent are summarized in Exp. 1, Table II, and it is evident that although there was preferential precipitation using WAB/Not anti-D23 serum (75·0 ± 9·7%) the non-specific precipitation by normal WAB/Not serum or WAB/Not anti-Mc7 serum was unacceptably high (51·5 ± 4·3 and 66·3 ± 4·0% respectively). Comparable levels of precipitation were also achieved using antigen preparations labelled with specific activities over the full range tested of 0·1 to 15 $\mu$Ci/$\mu$g protein. Clearly, denaturation and aggregation of the anti-genic preparation and/or chemical damage to the antigenic determinant may have accounted for these findings, and alternative radiolabelling procedures were adopted in order to minimize these problems.

By introducing $^{125}I$ into hepatoma D23 antigen by coupling $^{125}I$-tyrosine ($^{125}I$-Tyr) to protein with the bifunctional reagent I-ethyl-3(3-dimethylaminopropyl)-carbodiimide, it was attempted to avoid possible chemical inactivation of antigen following oxidation of tyrosine with chloramine T. In co-precipitation tests, 39·6 ± 8·9% of $^{125}I$-Tyr-coupled D23 antigen was precipitated using WAB/Not anti-D23 serum, compared with 10·7 ± 2·0% with normal WAB/Not serum ($P < 0{\cdot}01$) and 10·1 ± 0·7% with WAB/Not anti-Mc7 serum ($P < 0{\cdot}005$; Exp. 2, Table II).

Next, it was attempted to protect the antigenic determinant during radio-iodination by reacting immunoadsorbent-bound antigen with Na $^{125}I$, using a modified chloramine T procedure. After removal of $^{125}I$ by washing, the radio-labelled antigen was dissociated from the solid phase with 3M NaSCN, and this material (referred to as "indirect $^{125}I$-labelled" D23 antigen) was analysed in the co-precipitation test. In Table II, the results of 3 separate assays using this type of antigen are presented in Exps 3–5. In each experiment, the precipitation of antigen with WAB/Not anti-D23 serum

TABLE II.—*Co-precipitation of $^{125}I$-labelled tumour antigens*

| Exp. No. | Antigen* | % Coprecipitated by WAB/Not serum (mean ± s.d.)† | | | |
|---|---|---|---|---|---|
| | | Anti-D23 | Anti-D192 | Anti-Mc7 | Normal serum |
| | | NS (Anti-D23 vs Anti-Mc7) | | | < 0·05‡ (Anti-Mc7 vs Normal serum) |
| 1 | Direct $^{125}I$-labelled D23 antigen | 75·0 ± 9·7 | N.D.§ | 66·3 ± 4·0 | 51·5 ± 4·3 |
| | | < 0·05 (Anti-D23 vs Normal serum) | | | |
| | | < 0·005 (Anti-D23 vs Anti-Mc7) | | | NS (Anti-Mc7 vs Normal serum) |
| 2 | $^{125}I$-Tyrosine-coupled D23 antigen | 39·6 ± 8·9 | N.D. | 10·0 ± 0·7 | 10·7 ± 2·0 |
| | | < 0·01 (Anti-D23 vs Normal serum) | | | |
| | | < 0·05 (Anti-D23 vs Anti-D192) | NS (Anti-D192 vs Normal serum) | | |
| 3 | Indirect $^{125}I$-labelled D23 antigen | 44·8 ± 11·6 | 12·9 ± 5·2 | N.D. | 6·4 ± 4·2 |
| | | < 0·01 (Anti-D23 vs Normal serum) | | | |
| | | NS (Anti-D23 vs Anti-D192) | NS (Anti-D192 vs Normal serum) | | |
| 4 | Indirect $^{125}I$-labelled D23 antigen | 17·2 ± 8·4 | 8·3 ± 5·6 | N.D. | − 1·0 ± 5·9 |
| | | < 0·05 (Anti-D23 vs Normal serum) | | | |
| | | < 0·05 (Anti-D23 vs Anti-D192) | NS (Anti-D192 vs Normal serum) | | |
| 5 | Indirect $^{125}I$-labelled D23 antigen | 56·4 ± 18·7 | 16·4 ± 6·1 | N.D. | 17·4 ± 6·5 |
| | | < 0·05 (Anti-D23 vs Normal serum) | | | |

* Prepared as in Materials and Methods.
† Results of each experiment are expressed as mean of 3 determinations.
‡ Probabilities ($P$) calculated by Student's $t$ test.
§ Not done.

TABLE III.—*Radioimmunoadsorbent chromatography of $^{125}$I-labelled tumour antigens*

| Antigen | % Bound/eluted (mean ± s.d.) Anti-D23[1] | n | Anti-D192[2] | n | % Specific binding[3] | Specific ratio[4] |
|---|---|---|---|---|---|---|
| Direct $^{125}$I-labelled D23 antigen | 5·0 ± 2·5 | 3 | 5·0 ± 3·0 | 3 | 0 | 1·0 |
| $^{125}$I-Tyrosine-coupled D23 antigen | 20·0 ± 10·3 | 3 | 6·4 | 1 | 13·6 | 3·1 |
| Indirect $^{125}$I-labelled D23 antigen | 32·5 ± 5·7 | 4 | 5·8 | 1 | 26·7 | 5·6 |
| Indirect $^{125}$I-labelled D192A antigen[5] | 5·5 | 1 | 14·8 | 1 | 9·3 | 2·6 |
| Indirect $^{125}$I-labelled Sp4 antigen[6] | 2·6 | 1 | 6·7 | 1 | — | — |

[1] Sepharose-4B-linked WAB/Not anti-D23 IgG.
[2] Sepharose-4B-linked WAB/Not anti-D192 IgG.
[3] % increase in binding of $^{125}$I-labelled antigen to its appropriate immunoadsorbent.
[4] Ratio of % binding to specific immunoadsorbent to % binding to irrelevant immunoadsorbent.
[5] D192A 3M extract was incubated with Sepharose-4B-linked WAB/Not anti-D192A IgG. After appropriate washing, the Sepharose pellet was labelled with $^{125}$I as described in the text.
[6] Partially purified Sp4 antigens were prepared by papain hydrolysis of extra-nuclear membranes (Baldwin *et al.*, 1973*b*) chromatography on Con A-Sepharose and immunoadsorption on Sepharose-linked WAB/Not anti-Sp4 IgG. Specifically eluted antigen from this adsorbent was re-applied to an identical immunoadsorbent and radiolabelled by the indirect method as described in the text.
n = Number of chromatographic separations.

was significantly higher than that obtained using normal WAB/Not serum, and in Experiments 3 and 5 the increased precipitation with anti-D23 serum was statistically significantly greater than that determined using an irrelevant syngeneic immune serum (WAB/Not anti-D192 serum).

The results of these co-precipitation tests indicate that preferential reactivity of labelled D23 antigen with D23-immune serum compared with normal rat serum or an irrelevant immune serum, may be demonstrated, although non-specific interactions with rat sera may lead to high background precipitation (*e.g.* Exp. 1, Table II). The three types of radiolabelled antigen preparation were, therefore, assayed for their capacity to rebind to hepatoma D23 immune serum IgG coupled to Sepharose-4B beads. Using this procedure, nonspecific adsorption could be reduced by washing the immunoadsorbent with 0·1M glycine-NaOH, 0·5M NaCl buffer (pH 9·0) (Zöller & Matzku, 1976) and possible decreases in the affinity of labelled antigen for syngeneic antibodies following the secondary reaction with a precipitating antiglobulin reagent could be avoided. A total of 19 immunoadsorbent columns were prepared, and the results in Table III demonstrate that hepatoma D23 antigen directly radioiodinated by the chloramine T method failed to rebind to Sepharose-4B-immobilized WAB/Not anti-D23 serum IgG, and also did not bind to immobilized IgG from an irrelevant syngeneic immune serum (WAB/Not anti-D192 serum) indicating substantial if not total loss of antigenic activity. Hepatoma D23 antigen labelled with $^{125}$I-Tyr reacted preferentially with immobilized antibody from WAB/Not anti-D23 serum, 20·0% being retained in the solid phase, compared with only 6·4% bound to an irrelevant syngeneic immune serum IgG (Table III). 32·5% of indirectly labelled D23 antigen was retained upon the relevant immunoadsorbent, compared with only 5·8% bound to the irrelevant anti-D192 immunoadsorbent, giving a specific binding ratio of 5·6 (Table III).

The specificity of these interactions was further analysed using antigen preparations isolated from the rat hepatoma D192 and rat mammary carcinoma Sp4 using the same procedure as for hepatoma D23 antigen (Table I). Elevated binding of indirect $^{125}$I-labelled D192 antigen was detected (14·8% binding to D182-immune IgG compared with 5·5% binding to D23-immune IgG) and the $^{125}$I-labelled Sp4 antigen failed to bind to either immunoadsorbent (Table III).

One interpretation of these findings is that radiolabelling of purified, papain-solubilized hepatoma D23 antigen modifies its reactivity with tumour-immune

FIGURE.—Radioimmunoadsorbent chromatography of $^{125}$I-Tyr-coupled hepatoma D23 antigen on Sepharose-4B-linked WAB/Not anti-D23 IgG. Column A dimensions = 26 x 50 mm, column B dimensions = 450 x 9 mm. The sample applied to Column B was the unbound fraction recovered from Column A.

serum, by reducing its affinity for syngeneic antibodies. This is supported by the radioimmunoadsorbent chromatography experiments shown in the Figure. When $^{125}$I-Tyr-coupled hepatoma D23 was applied to a conventional immunoadsorbent column of Sepharose 4B-linked WAB/Not anti-D23 IgG (26 mm in length x 50 mm in diameter, Column A) most of the labelled protein passed through the column as a single peak. However, if the affinity substrate was packed into a long, narrow column (450 mm in length x 9 mm in diameter, Column B) the unbound fraction was found to contain a proportion of antigen which was retarded by interaction with immobilized antibody. This effect was not found using an irrelevant immunoadsorbent (Sepharose-4B-conjugated WAB/Not anti-D192 serum IgG) and was not chromatographic, as distinct from an immunoaffinity, phenomenon, since labelled antigen eluted from primary amine-linked Sepharose-4B as a single peak. These findings further confirm the proposal that radiolabelled hepatoma D23 antigen exhibits a reduced affinity for syngeneic antibodies, since unlabelled antigens have been found to be retained on their appropriate immunoadsorbent, and dissociating conditions are required to effect their release.

### DISCUSSION

Many attempts have now been made to purify tumour-specific antigens from what are initially highly heterogeneous extracts, the eventual aim of these studies being to obtain a chemical definition of the specificity of these determinants. At the present time the major problem limiting progress in this area is the lack of sensitive and quantitative immunoassays for soluble tumour-specific antigens. However, the early work of Thomson *et al.* (1973) using a solid-phase radioimmunoassay for antigen in the serum of rats bearing grafts of a 3-methylcholanthrene-induced sarcoma suggests that it is possible to develop cell-free immunoassays of the required sensitivity, though in more recent studies Thomson *et al.* (1976) have returned to indirect membrane-immunofluoresence tests to monitor the purification of sarcoma-associated antigens.

As an alternative, co-precipitation tests may provide a worthwhile approach for the quantitation of soluble tumour antigens. This procedure has already been used by Wolf *et al.* (1976) for the estimation of circulating antigen in mice bearing the SL2 lymphoma, but as the authors pointed out, the assay could only be regarded as semi-quantitative because of the relatively high backgrounds and interference with normal serum constituents.

Using the 3-methylcholanthrene-induced murine sarcoma, Meth A, Natori *et al.* (1978) have largely eliminated non-specific immunoprecipitation of labelled antigen by pre-absorbing the anti-serum

*in vivo*. It should be emphasized however that the antiserum used was a heterologous serum prepared against a partially purified Meth A antigen fraction, and so determinants other than those recognized by syngeneic immune sera may be detected. An additional complication arises since other studies have established that although syngeneic anti-Meth A sera are specifically cytotoxic for Meth A cells (DeLeo *et al.*, 1977) syngeneic antisera are also reactive in radioimmunoprecipitation tests with a common, transformation-related antigen in chemically induced sarcomas and other transformed cell lines (DeLeo *et al.*, 1979). This information is in itself of interest in defining the transformation process, though from the present investigation it would appear that this transformation-related protein, p53, is not being detected in the rat hepatoma model, since the labelled antigens retain individually distinct specificity in their reaction with syngeneic antibody. Thus, a major conclusion from the results in this report is that serological reactivity may be retained in radioiodinated antigen preparations, though care must be taken in choosing the method of radiolabelling. In particular, the mild oxidizing agent chloramine T would appear to be particularly deleterious to these antigens, which might imply that tyrosine is a component of, or a residue in the immediate environment of, the antigenic determinant itself. The loss of affinity of radiolabelled hepatoma D23 antigen for appropriate specific antibodies is reflected in the radioimmunoadsorption chromatography experiments shown in the Figure. Although immunoadsorption chromatography on insolubilized antibodies of very low affinity has been established as an effective procedure for the large-scale isolation of tumour-associated antigens (Ruoslahti, 1978) this particular result has other significance in terms of the development of a cell-free radioimmunoassay, since the affinity constant for the binding of unlabelled, inhibitor antigen will be higher than that for its radiolabelled counterpart. This would then indicate that, for a cell-free radioimmunoassay for hepatoma D23 antigen, it may be more appropriate to use metabolically radiolabelled antigens, or alternatively an unlabelled reference soluble antigen must be used in conjunction with a secondary radioactive indicator. Both approaches are currently being explored and, for the latter, efforts are being directed towards the development of an assay with which the specific binding of purified hepatoma D23-specific antigen to immobilized antibody is revealed by the uptake of $^{125}$I-labelled Concanavalin A to the glycosylated moiety of the antigenic glycopeptide.

This study was supported by the Cancer Research Campaign and by a Government Equipment Grant obtained through the Royal Society. D. Hannant was supported by NCI Contract Number NO1-CB-74167.

The authors acknowledge with thanks the skilful technical assistance of Mrs J. E. Bullock, Miss S. Crosdale and Mrs J. Manning. Mrs M. E. Addison and the staff of the Cancer Research Campaign Animal Unit are thanked for the provision and maintenance of animals.

### REFERENCES

AL-SHEIKLY, A. W., EMBLETON, M. J. & PRICE, M. R. (1979) Detection of tumour specific antigens and alloantigens using a radioisotopic antiglobulin assay. In *Proc. Vth Meeting Eur. Assoc. Cancer Res.* Ed. Letnansky. Amsterdam: Kugler Medical Publications. (In press.)

BALDWIN, R. W. (1973) Immunological aspects of chemical carcinogenesis. *Adv. Cancer Res.*, **18**, 1.

BALDWIN, R. W., EMBLETON, M. J. & PRICE, M. R. (1973*a*) Inhibition of lymphocyte-cytotoxicity for human colon carcinoma by treatment with solubilized tumour membrane fractions. *Int. J. Cancer*, **12**, 84.

BALDWIN, R. W., HARRIS, J. R. & PRICE, M. R. (1973*b*) Fractionation of plasma membrane-associated tumour specific antigen from an aminoazo dye-induced rat hepatoma. *Int. J. Cancer*, **11**, 385.

BOWEN, J. G. & BALDWIN, R. W. (1979) Tumour antigens and alloantigens. I. Cross-reactivity of rat tumour-specific antigens with normal alloantigens of the host strain. *Int. J. Cancer*, **23**, 826.

DELEO, A. B., SHIKU, H., TAKAHASHI, T., JOHN, M. & OLD, L. J. (1977) Cell surface antigens of chemically-induced sarcomas in the mouse. *J. Exp. Med.*, **146**, 720.

DELEO, A. B., JAY, G., APPELLA, E., DUBOIS, G. C., LAW, L. W. & OLD, L. J. (1979) Detection of a transformation-related antigen in chemically induced sarcomas and other transformed cells of the mouse. *Proc. Natl Acad. Sci.*, **76**, 2420.

McConahey, P. J. & Dixon, F. J. (1966) A method of trace iodination of proteins for immunological studies. *Int. Arch. Allergy*, **29**, 185.

Moore, M. (1978) Antigens of experimentally-induced neoplasms: A conspectus. In *Immunological Aspects of Cancer*. Ed. Castro. Lancaster: Medical and Technical Publishing Co. Ltd. p. 15.

Natori, T., Law, L. W. & Appella, E. (1978) Immunochemical evidence of a tumor-specific surface antigen obtained by detergent solubilization of the membranes of a chemically induced sarcoma, Meth A. *Cancer Res.*, **38**, 359.

Phillips, D. R. & Morrison, M. (1971) Exposed protein on the intact human erythrocyte. *Biochemistry*, **10**, 1766.

Preston, V. E. & Price, M. R. (1977) Partial purification of a plasma membrane associated tumour specific antigen from a rat sarcoma by using immunoadsorbent column chromatography. *Biochem. Soc. Trans.*, **5**, 123.

Price, M. R. & Baldwin, R. W. (1977) Shedding of tumor cell surface antigens. In *Dynamic Aspects of Cell Surface Organization*. Eds. Poste & Nicholson. Amsterdam: Elsevier. p. 423.

Price, M. R., Moore, V. E. & Baldwin, R. W. (1979) Biochemical aspects of tumour specific antigens. In *Current Trends in Immunology*. Eds. Ferrone *et al.* New York: Garland Press, Inc. (In press.)

Robins, R. A. (1975) Serum antibody responses to an ascitic variant of rat hepatoma D23. *Br. J. Cancer*, **32**, 21.

Ruoslahti, E. (1978) Immunochromatography on insolubilised antibodies of very low affinity: Applications to immunoadsorbence of bovine $\alpha$-fetoprotein. *J. Immunol.*, **121**, 1687.

Thomson, D. M. P., Sellens, V., Eccles, S. & Alexander, P. (1973) Radioimmunoassay of tumour specific transplantation antigen of a chemically-induced rat sarcoma: Circulating soluble tumour antigen in tumour bearers. *Br. J. Cancer*, **28**, 377.

Thomson, D. M. P., Gold, P., Freedman, S. O. & Shuster, J. (1976) The isolation and characterization of tumor specific antigens of rodent and human tumors. *Cancer Res.*, **36**, 3518.

Wolf, A., Steele, K. A. & Alexander, P. (1976) Estimation in sera by radioimmunoassay of a specific membrane antigen associated with a murine lymphoma. *Br. J. Cancer*, **33**, 144.

Zöller, M. & Matzku, S. (1976) Antigen and antibody purification by immunoadsorption: Elimination of non-biospecifically bound protein. *J. Immunol. Meth.*, **11**, 287.


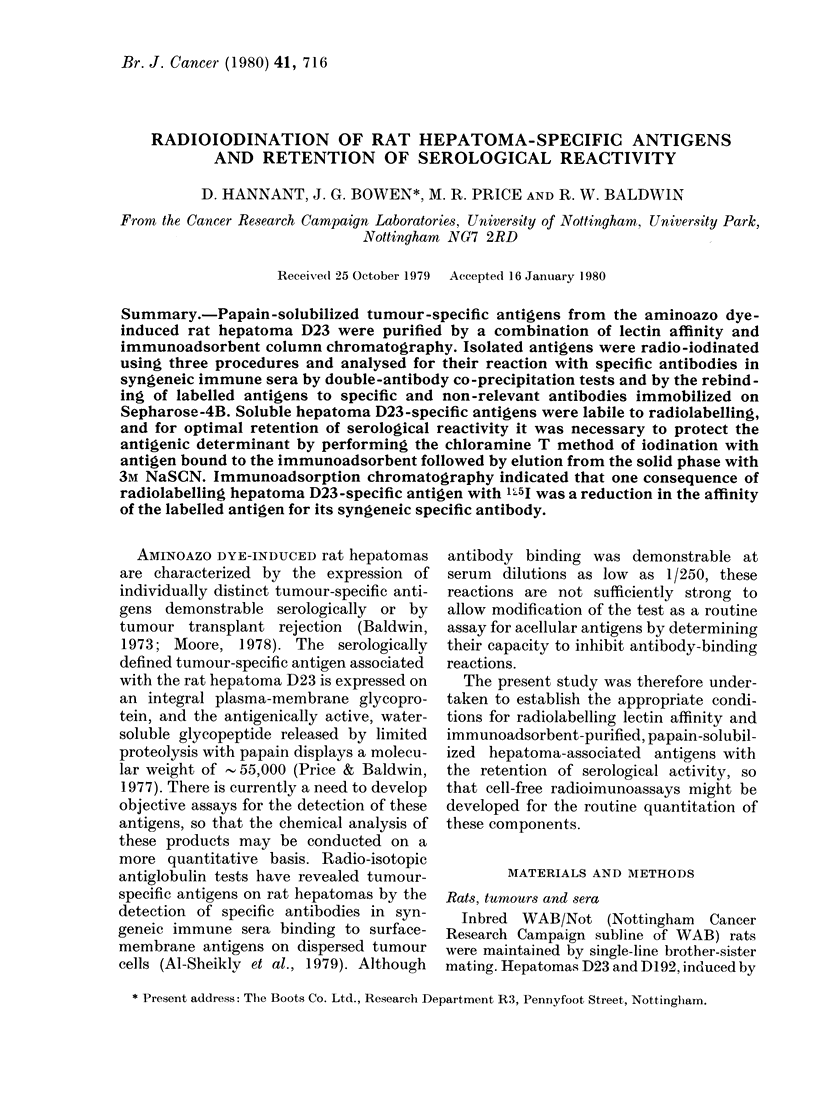

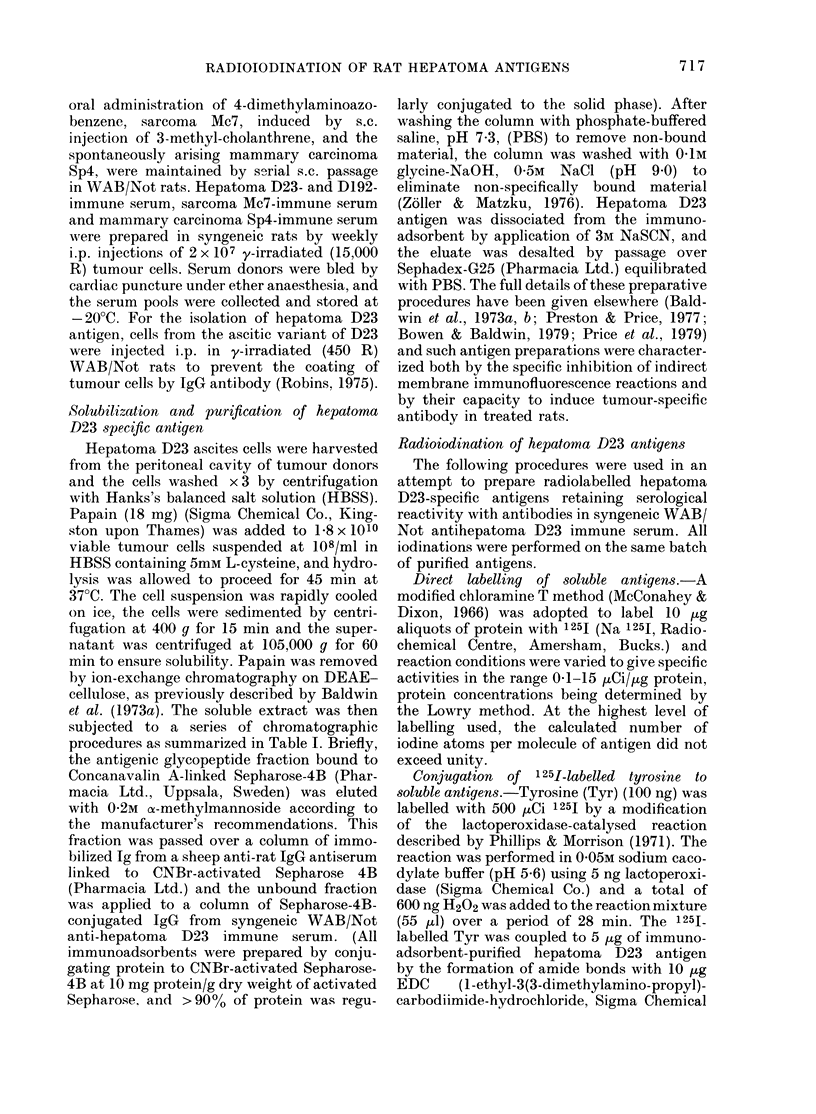

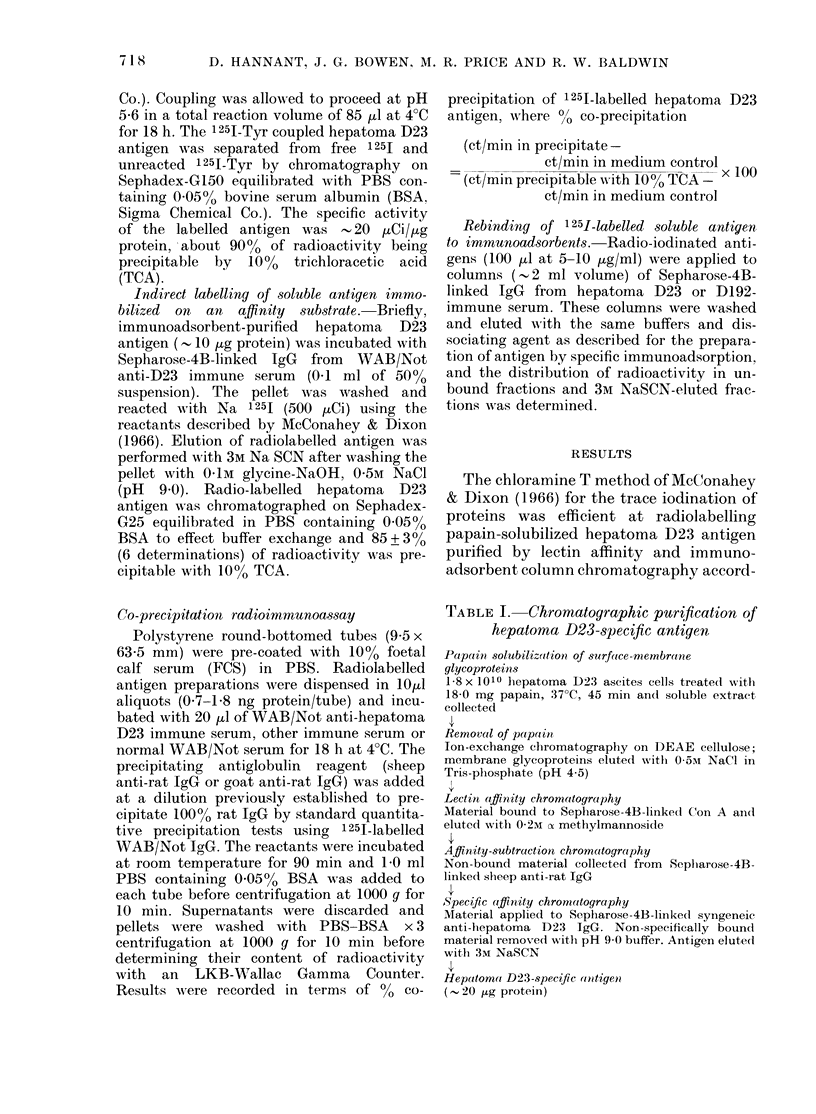

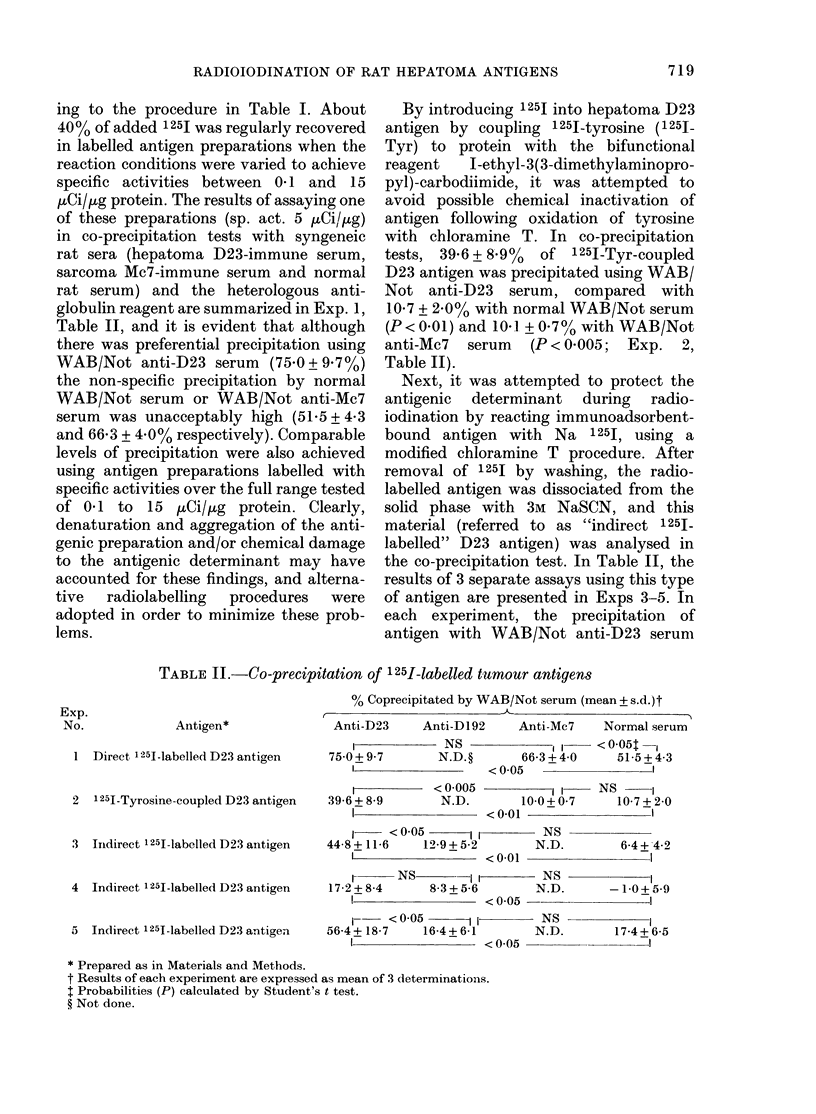

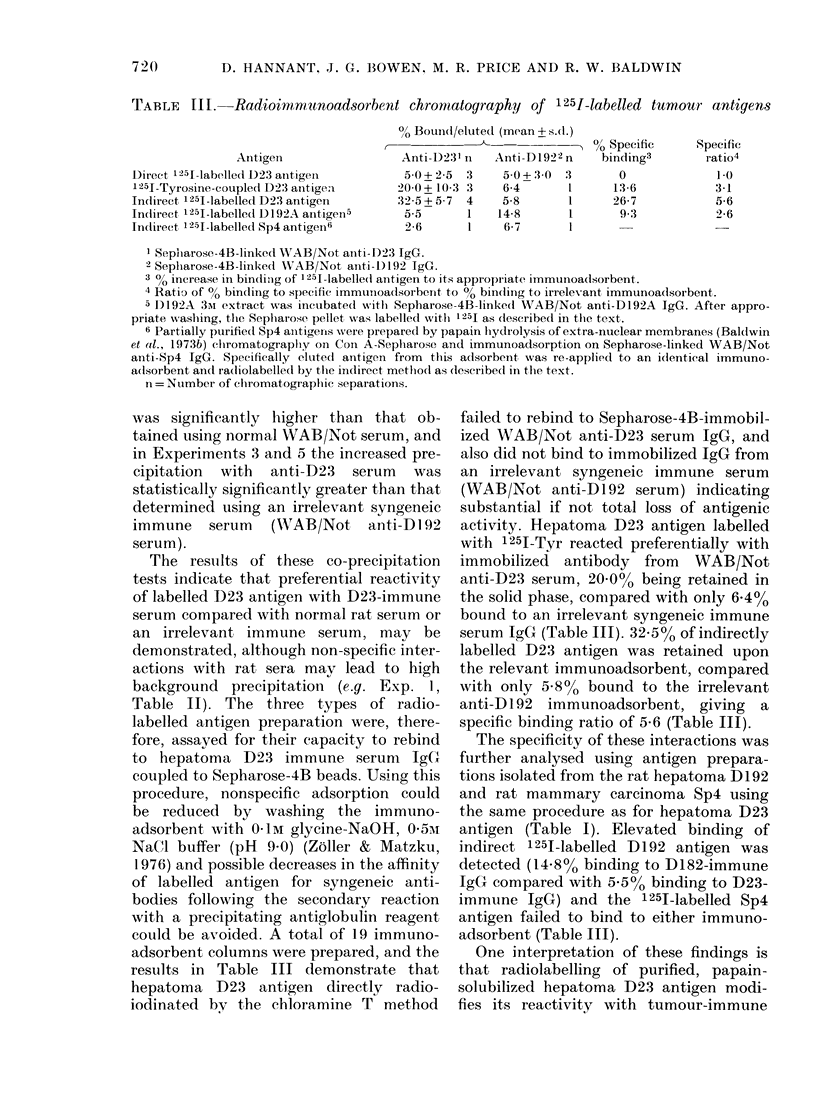

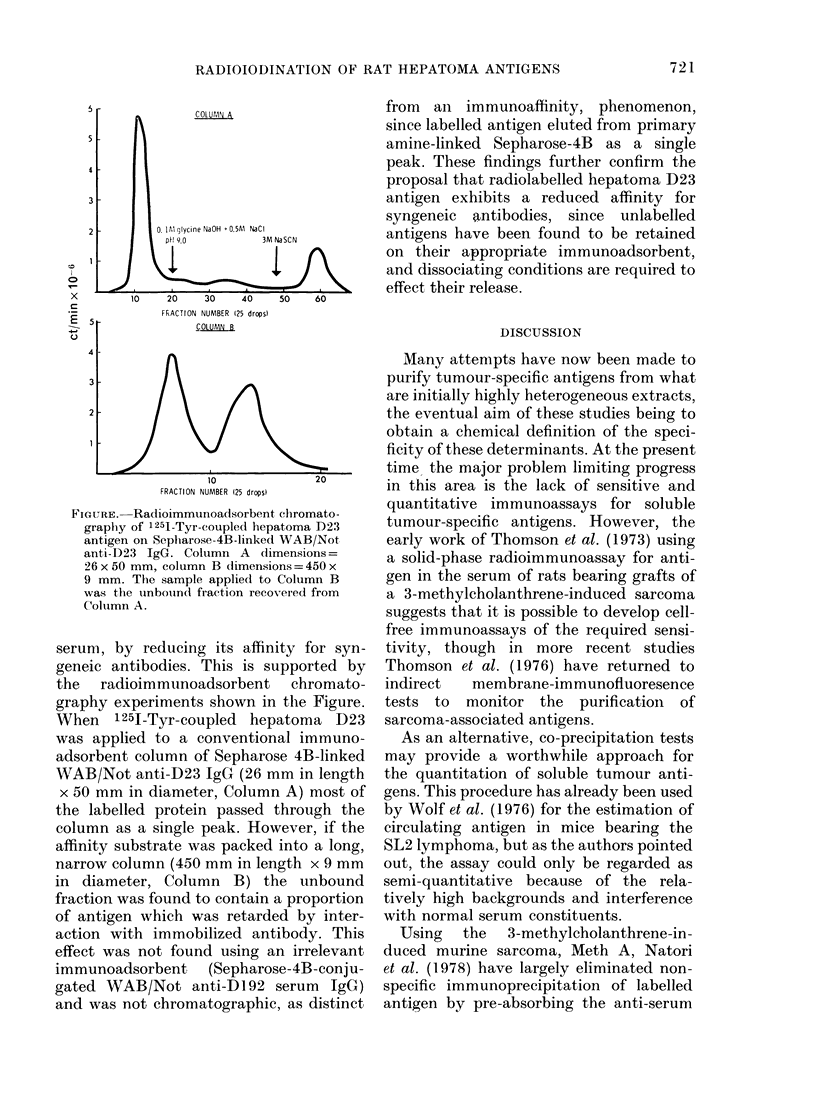

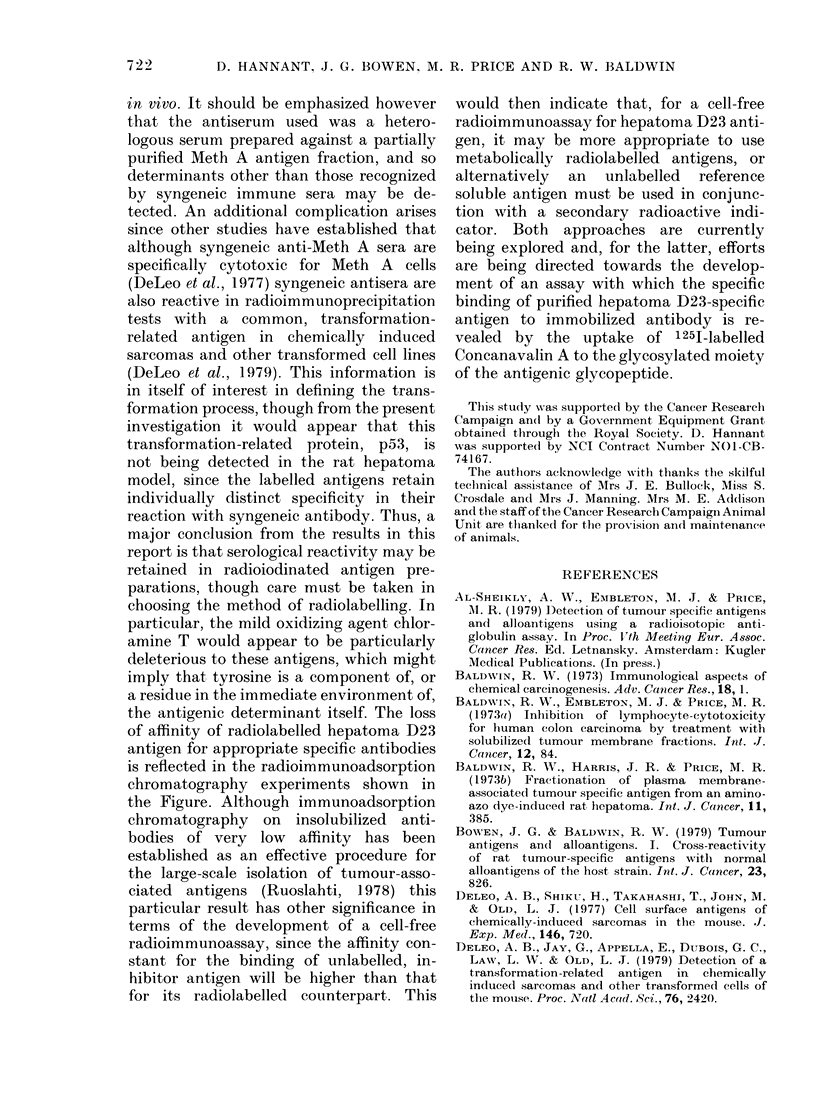

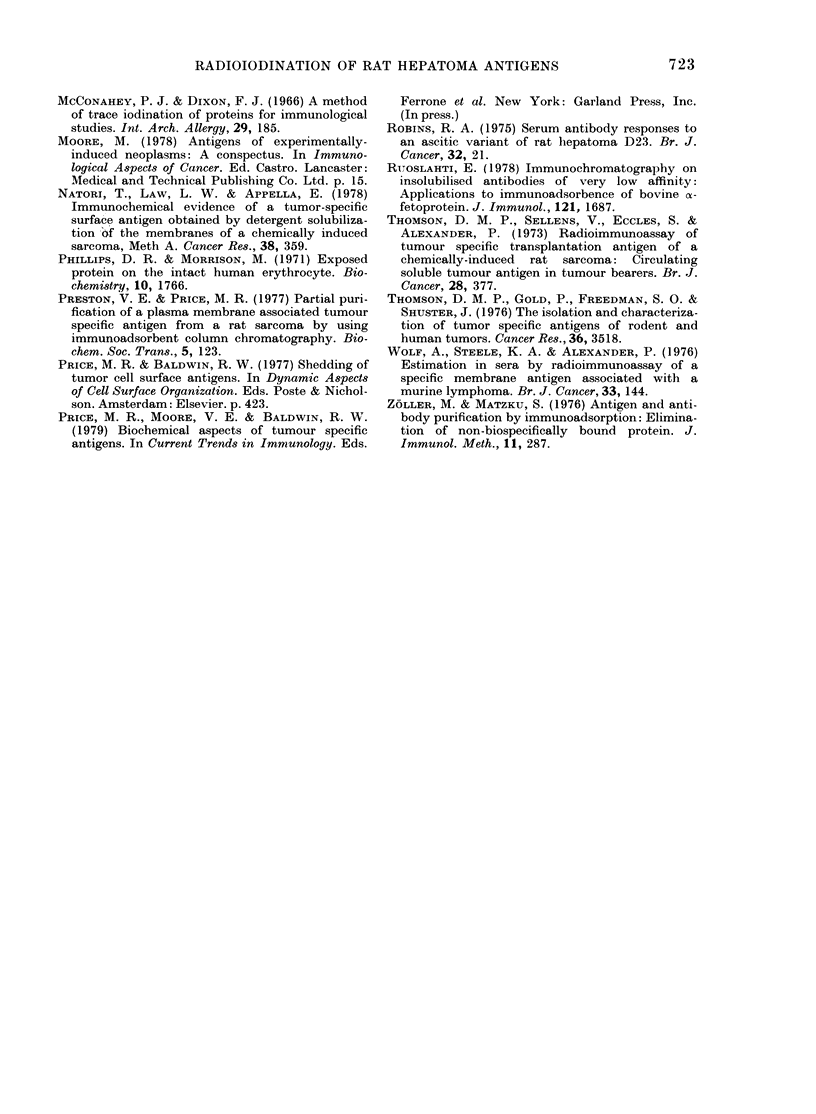


## References

[OCR_00821] Baldwin R. W., Harris J. R., Price M. R. (1973). Fractionation of plasma membrane-associated tumor-specific antigen from an aminoazo dye-induced rat hepatoma.. Int J Cancer.

[OCR_00828] Bowen J. G., Baldwin R. W. (1979). Tumour antigens and alloantigens. I. Relation of a rat tumour-specific antigen with normal alloantigens of the host strain.. Int J Cancer.

[OCR_00841] DeLeo A. B., Jay G., Appella E., Dubois G. C., Law L. W., Old L. J. (1979). Detection of a transformation-related antigen in chemically induced sarcomas and other transformed cells of the mouse.. Proc Natl Acad Sci U S A.

[OCR_00835] DeLeo A. B., Shiku H., Takahashi T., John M., Old L. J. (1977). Cell surface antigens of chemically induced sarcomas of the mouse. I. Murine leukemia virus-related antigens and alloantigens on cultured fibroblasts and sarcoma cells: description of a unique antigen on BALB/c Meth A sarcoma.. J Exp Med.

[OCR_00850] McConahey P. J., Dixon F. J. (1966). A method of trace iodination of proteins for immunologic studies.. Int Arch Allergy Appl Immunol.

[OCR_00860] Natori T., Law L. W., Appella E. (1978). Immunochemical evidence of a tumor-specific surface antigen obtained by detergent solubilization of the membranes of a chemically induced sarcoma, meth-A.. Cancer Res.

[OCR_00867] Phillips D. R., Morrison M. (1971). Exposed protein on the intact human erythrocyte.. Biochemistry.

[OCR_00872] Preston V., Price M. R. (1977). Partial purification of a plasma-membrane-associated tumour-specific antigen from a rat sarcoma by using immunoadsorbent column.. Biochem Soc Trans.

[OCR_00893] Robins R. A. (1975). Serum antibody responses to an ascitic variant of rat hepatoma D23.. Br J Cancer.

[OCR_00898] Ruoslahti E. (1978). Immunochromatography on insolubilized antibodies of very low affinity: application to immunoadsorbence of bovine alpha-fetoprotein.. J Immunol.

[OCR_00912] Thomson D. M., Gold P., Freedman S. O., Shuster J. (1976). The isolation and characterization of tumor-specific antigens of rodent and human tumors.. Cancer Res.

[OCR_00904] Thomson D. M., Sellens V., Eccles S., Alexander P. (1973). Radioimmunoassay of tumour specific transplantation antigen of a chemically induced rat sarcoma: circulating soluble tumour antigen in tumour bearers.. Br J Cancer.

[OCR_00918] Wolf A., Steele K. A., Alexander P. (1976). Estimation in sera by radioimmunoassay of a specific membrane antigen associated with a murine lymphoma.. Br J Cancer.

[OCR_00924] Zoller M., Matzku S. (1976). Antigen and antibody purification by immunoadsorption: elimination of non-biospecifically bound proteins.. J Immunol Methods.

